# The Primodos components Norethisterone acetate and Ethinyl estradiol induce developmental abnormalities in zebrafish embryos

**DOI:** 10.1038/s41598-018-21318-9

**Published:** 2018-02-13

**Authors:** Samantha Brown, Lucas Rosa Fraga, Gary Cameron, Lynda Erskine, Neil Vargesson

**Affiliations:** 1 School of Medicine, Medical Sciences and Nutrition, Institute of Medical Sciences, University of Aberdeen. Foresterhill, Aberdeen, AB25 2ZD UK; 2 School of Medicine, Medical Sciences and Nutrition, The Rowett Institute, University of Aberdeen. Foresterhill, Aberdeen, AB25 2ZD UK; 3 Present Address: Genetics Department, Biosciences Institute, Universidade Federal do Rio Grande do Sul, Porto Alegre, 91501-970 Brazil

## Abstract

Primodos was a hormone pregnancy test used between 1958–1978 that has been implicated with causing a range of birth defects ever since. Though Primodos is no longer used, it’s components, Norethisterone acetate and Ethinyl estradiol, are used in other medications today including treatments for endometriosis and contraceptives. However, whether Primodos caused birth defects or not remains controversial, and has been little investigated. Here we used the developing zebrafish embryo, a human cell-line and mouse retinal explants to investigate the actions of the components of Primodos upon embryonic and tissue development. We show that Norethisterone acetate and Ethinyl estradiol cause embryonic damage in a dose and time responsive manner. The damage occurs rapidly after drug exposure, affecting multiple organ systems. Moreover, we found that the Norethisterone acetate and Ethinyl estradiol mixture can affect nerve outgrowth and blood vessel patterning directly and accumulates in the forming embryo for at least 24 hrs. These data demonstrate that Norethisterone acetate and Ethinyl estradiol are potentially teratogenic, depending on dose and embryonic stage of development in the zebrafish. Further work in mammalian model species are now required to build on these findings and determine if placental embryos also are affected by synthetic sex hormones and their mechanisms of action.

## Introduction

Primodos (known as Duogynon in Germany) is a trade name of a hormonal-based pregnancy test composed of 10 mg of norethisterone acetate (NA), a synthetic progestogen, and 0.02 mg of ethinyl estradiol (EE), a synthetic oestrogen. Primodos was marketed in the UK between 1958 and 1978 as a method of testing for pregnancy, based on whether the woman menstruated after taking Primodos or not^1–3^. Its mechanism of action was simple. It causes a rapid spike in the levels of progesterone. If a woman is pregnant she will have higher levels of progesterone, which maintain pregnancy normally. It was presumed that the increase in progesterone would be balanced out by the normally higher levels of pregnancy induced progesterone. If she was not pregnant, then the rapid spike in progesterone would be lost, and this mimics the end of the menstrual cycle, resulting in a small bleed. Intake of Primodos during pregnancy has been potentially linked to a range of birth defects including neural tube closure defects, cleft lip and palate, limb defects and cardiovascular defects^2,4–10^. Several epidemiological studies have provided support for a potential link between Primodos, as well as other hormone pregnancy tests, and birth defects^2,7,8,11–14^. Further support for the idea that Primodos is teratogenic has come from experiments in animal models, demonstrating that progestins and synthetic oestrogens induce brain malformations, embryonic death and genital malformation in mice foetuses^15–17^, rats^18^ and embryonic death and abortion in rhesus monkey, Cynomolgus monkey and baboons^17,19^. However, other epidemiological studies have failed to find a link between the use of hormone pregnancy test, such as Primodos, and causation of birth defects^9,20^. In addition, some experimental studies found no congenital abnormalities in rats and rabbits exposed to progestin’s and synthetic oestrogens^17,21,22^. Moreover, studies looking at external genitalia malformations caused by exposure to sex hormones in the first trimester suggest there was no causal association^23^. Based on the current evidence it is far from clear whether exposure to Primodos or its components has the potential to cause embryonic or foetal damage. Primodos is no longer on the market but its components, alone or in combination, are still found in many medications today. Examples of their use today include hormone replacement therapy, secondary amenorrhea and period delay as well as emergency contraception (ie: morning after pill) and in some contraceptive preparations but at much smaller dosages than Primodos was used at (less than 0.5 mg)^17,24–28^. Today the packaging of drugs containing these components carries warning signs they should not be used in pregnancy as there is a risk to the unborn child^27,28^. However, whether these drugs are teratogenic remains unclear.

The zebrafish embryo has become increasingly popular in drug screening assays due to its rapid development, optical transparency and the ability to visualise and follow development live and *in vivo*^29^. Indeed, many drugs have actions in zebrafish embryos that are similar to actions in mammalian species including humans. For example, thalidomide exposure causes damage in zebrafish embryos in similar or equivalent tissues damaged in humans following thalidomide exposure^30–33^. Furthermore, zebrafish embryos are becoming increasingly popular to screen compounds to identify lead compounds that could be used for further analysis in mammalian species and/or to determine if compounds may have harmful effects^29,30,34–37^.

Using a combination of *in vivo* and *in vitro* assays for teratogenesis, angiogenesis, cell death, cell proliferation and neurotoxicity the effects of Norethisterone acetate and Ethinyl estradiol (in a ratio similar to that seen in Primodos) was analysed in zebrafish embryos, mouse retinal explants and HUVEC cell culture. We found that these compounds had a dose and time dependent effect on zebrafish embryo development, affecting eyes, fins, the spine, overall length of the embryo, vascular development and nerve growth and defasciculation. Moreover, our results demonstrate that the effect of these compounds depends on the developmental stage of the embryos. Its actions on the embryo are rapid and that the amount of drug that enters the embryo accumulates for 24 hr. Our results indicate that direct exposure to a high dose of a mixture of Norethisterone acetate and Ethinyl estradiol induces morphological defects in developing zebrafish embryos.

## Results

### A Norethisterone acetate (NA) and Ethinyl estradiol (EE) mixture impairs zebrafish development and survival in a dose responsive manner

**Figure 1:**
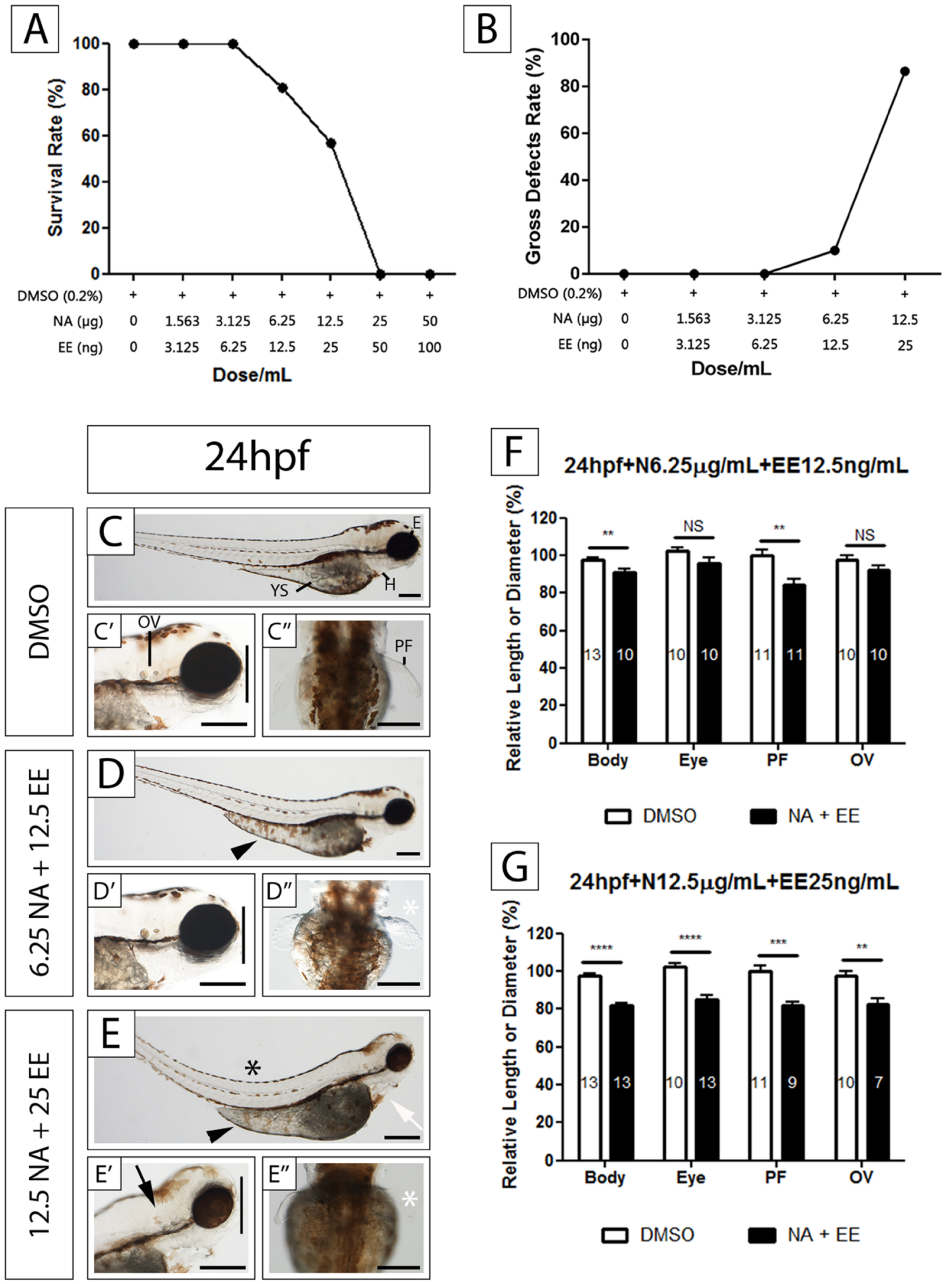
Effects of exposure to the NA/EE Mixture upon Survival and Development of Zebrafish Embryos Are Time and Dose Sensitive Zebrafish embryos at 24 hpf were treated with either DMSO, NA 6.25 μg/mL + EE 12.5 ng/mL or NA 12.5 μg/mL + EE 25 ng/mL, incubated until 96 hpf and overall body size, PF, OV and eye size measured. (**A**) The survival rate decreases as the dose increases. Survival rate starts to decrease at a concentration of NA 6.25 µg/mL + EE 12.5 ng/mL. (**B**) The rate of gross defects occurrence increases as the dose increases. Embryos start to present defects at a concentration of NA 6.25 µg/mL + EE 12.5 ng/mL (**C**–**G**) NA and EE mixture causes reduction of body size, PF, OV and eye size in a dose-dependent manner. Damage caused by the drugs is less severe in embryos treated with lower dose (**D–D”**) when compared to DMSO controls (**C–C”** and **F**). NA: Norethisterone acetate. EE: Ethinyl Estradiol. H: heart; YS: yolk sac; OV: otic vesicle; PF: pectoral fin. Black arrowhead indicates yolk sac and extension oedema. Black arrow indicates otic vesicle is smaller. Black asterisk denotes bent spine. White asterisk denotes reduced (D”) or missing pectoral fin (E”). White arrow indicates pericardial oedema. Vertical black line compares the eye diameter between NA/EE-mixture and DMSO treated embryos in C’, D’ and E’, and indicates diameter of the eye is altered in NA/EE-treated embryos. Relative length: compared to WT. Statistical significance was analysed using Student t-test. Graphs represent mean ± SEM. **p < 0.01, ***p < 0.001 and ****p < 0.0001. Scale bars: 250 µm.

Primodos, which is no longer made, was composed of 10 mg Norethisterone acetate (NA) and 0.02 mg Ethinyl estradiol (EE). We therefore screened the effect on zebrafish embryos of a NA/EE-mixture at a ratio of 500:1 (NA:EE; equivalent ratio of their formulation in Primodos) at a range of concentrations. In the majority of experiments, drugs were added at 24 hpf, and the embryos fixed 6 to 72 hrs later (n ≥ 15 per treatment; Fig. 1A,B). This developmental time point is the period where most tissues and organs are rapidly developing and has been used in previous work in the lab analysing drug actions upon embryogenesis^30,32,34,35,37–39^. Moreover, this time period relates to approximately weeks 6–10 in human embryo development when hormonal pregnancy tests were likely to be used.

Lower doses of the NA/EE-mixture, 1.5 µg/mL NA + 3.125 ng/mL EE and 3.125 µg/mL NA + 6.25 ng/mL EE were non-lethal (n = 15), and had no obvious effect on embryonic development (Fig. 1A,B). However, as the dose increased we saw malformations in a dose-dependent manner and higher doses were 100% embryolethal. We observed some embryonic defects (approx. 20%) and a small increase in embryonic death (approx. 10%) at 6.25 µg/mL NA + 12.5 ng/mL EE (n = 62). At 12.5 µg/mL NA + 25 ng/mL EE (n = 91) markedly more embryos displayed damage (approx. 92% of all embryos had damage**)** and almost 50% of the embryos died (Fig. 1A,B). Doses of 25 µg/mL NA + 50 ng/mL EE or higher caused 100% lethality (n = 23) (Fig. 1A,B).

To further analyse the dose-dependent effect of NA/EE-mixture upon embryogenesis, we measured the overall size of the embryos, pectoral fin, otic vesicle and eye size of drug treated embryos and compared to DMSO controls. Lower doses of 1.5 µg/mL NA + 3.125ng/mL EE and 3.125 µg/ml + NA/6.25 ng/ml EE had no significant effect on the size of any of these structures. However, in embryos exposed to NA 6.25 μg/mL NA and 12.5 ng/mL EE the length of the pectoral fins and overall body length were decreased significantly (Fig. 1C–G). At a concentration of 12.5 μg/mL NA + 25 ng/mL EE the eye and otic vesicle were also decreased significantly in size. At this concentration, the embryos also exhibited a range of other malformations including bent spine, smaller overall size, pericardial and yolk sac oedema and oedematous yolk sac extension (Fig. 1E,G). Measurement of embryo length, pectoral fin size, otic vesicle size and eye diameter demonstrated 15–17% reduction in size of these parameters compared to the DMSO controls (Fig. 1C–C”, E–E” and G). This data demonstrates that the NA/EE-mixture impairs embryonic development in a dose dependent manner.

### Embryos at earlier developmental stages are more sensitive to the NA/EE-mixture

**Figure 2:**
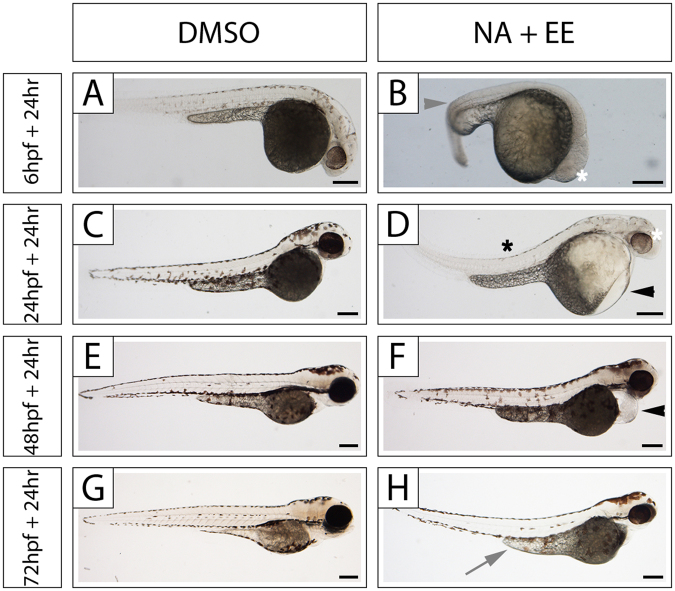
The NA/EE-mixture induces morphological damage in zebrafish embryos at 6 hpf, 24 hpf, 48 hpf and 72 hpf in a stage-sensitive manner. Embryos at 6 hpf, 24 hpf, 48 hpf and 72 hpf were treated with either DMSO or NA/EE-mixture (NA 12.5 μg/mL + EE 25 ng/mL), incubated for 24 hours then fixed and imaged. (**A**–**H**) Embryos at 6 hpf (**A**,**B**), 24 hpf (**C**,**D**), 48 hpf (**E**, **F**), 72 hpf (**G,H**) treated with DMSO (**A**,**C**,**E**,**G**) or NA/EE-mixture (**B,D,F,H**; n > 15 for all time points and conditions). Note: twisted spine (grey arrowhead); smaller eye (white asterisk); yolk sac anomalies/oedema (grey arrow), bent spine (black asterisk) and pericardial defect (black arrowhead). Scale bars: 250 µm.

When applied at 24 hpf the 12.5 μg/mL NA + 25 ng/mL EE mixture gave a 57% survival rate with the majority (92%) of embryos presenting defects (Fig. 1). We therefore focused our further experiments on this dose. First, we asked if this drug combination had a differential effect on embryos at different stages of development. We exposed 6 hpf, 24 hpf, 48 hpf and 72 hpf embryos to 12.5 μg/mL NA + 25 ng/mL EE and analysed the embryos 24 hrs later (Fig. 2). We found that early embryos are more severely affected than later stage (older) embryos (Fig. 2). Thus, embryos exposed at 6 hpf exhibited severely malformed tails and bent spines, malformed pericardial sacs, yolk sac damage/oedema and very small eyes, whereas embryos exposed at 48 hpf and at 72 hpf, had less severely bent spines, mild pericardial defects and their eyes and otic vesicle appeared to be normal; the only consistently observable issue was an oedematous yolk sac. This indicates that early stage embryos are more sensitive to the NA/EE-mixture than later stage embryos.

### Exposure to the NA/EE-mixture causes rapid morphological damage

**Figure 3:**
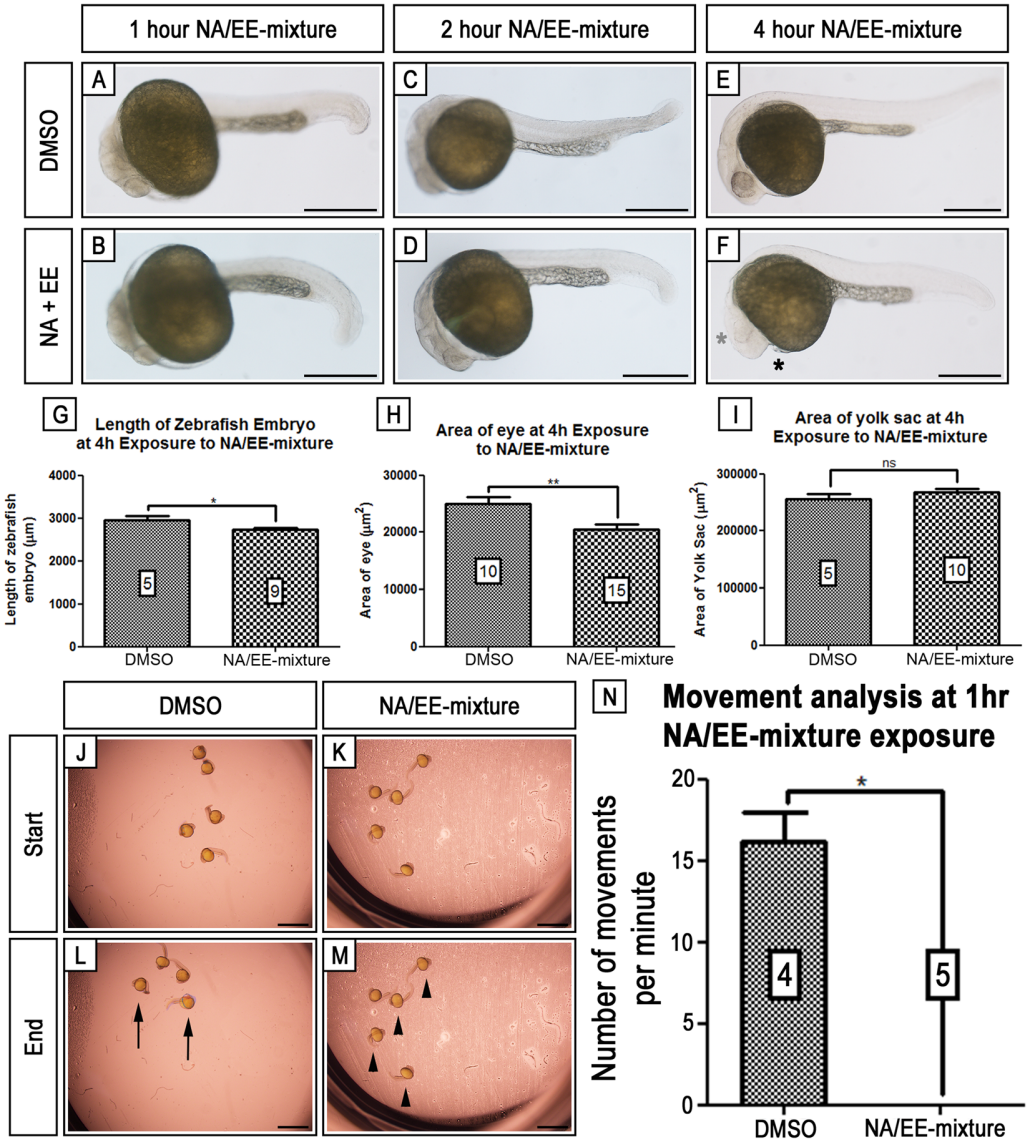
3 Embryonic damage is seen within 4 hrs of NA/EE-mixture exposure and embryonic movement is stunted from 1 hr of exposure. Zebrafish embryos at 24 hpf were treated with either DMSO (**A**,**C**,**E**) or NA/EE-mixture (**B**,**D**,**F**) for 1 hr (**A**,**B**), 2 hr (**C**,**D**) or 4 hr (**E**,**F**) before fixation. Overall body length, eye area and yolk sac area were measured. Embryos begin to show heart defects (n = 2/5) (black asterisk) and smaller body length (**G**) and eye area and pigment (**H**) (grey asterisk) by 4 hr exposure. At 4 hr, there is no change in the area of the yolk sac between DMSO control and NA/EE-mixture treated embryos (**I**). At 1 hr exposure, it was noted that there was a drastic decrease in movement in NA/EE-mixture treated embryos. This was determined using time lapse recording of embryos for 2 minutes and counting the number of times each embryo moved. The lack of movement can be visualised by comparing the starting and finishing positions of the embryos (**J**–**M**). In the DMSO treated well, there has been clear movement between 0 min (**J**) and 2 min (**L**), with one embryo having moved out of frame. Black arrows denote embryos in different positions. In comparison, the NA/EE-mixture treated embryos can be observed to have remained in the same position between 0 min (**K**) and 2 min (**M**). Black arrowheads show embryos in same position. The average number of movements recorded for DMSO treated embryos was 16 per minute compared to the average 0 per minute in the NA/EE-mixture treated embryos (**N**).

Next, we determined how quickly the NA/EE-mixture induces embryonic damage. We exposed 24 hpf embryos for differing time periods to the drug from 1 hr to 24 hrs (Fig. 3A–F). We found that the drug mixture acts rapidly and that the first distinct morphological damage was evident from 4 hrs after NA/EE-mixture application (Fig. 3G,I). We found eye size and body length were significantly reduced by 4 hrs of incubation. The forming heart also showed changes by 4 hrs of incubation in a subset of embryos (n = 2/5; Fig. 3F). Other tissues, such as the yolk sac, appeared unaffected at this timepoint (Fig. 3I). We also noted that movement of the embryos was inhibited after just 1 hr exposure to the drug mixture (Fig. 3J–N). We filmed embryos over a 2 minute period and counted the number of tail movements. Treated embryos made no tail movements (Fig. 3N) and were in the same position at the end of filming as at the start of the filming (Fig. 3K,M). In contrast control embryos were constantly moving and were in very different positions in the well by the end of filming (Fig. 3J,L,N). This inhibition of movement was also observed at all timepoints assessed up to and including 4 hrs of exposure (n ≥ 5). This indicates that the NA/EE-mixture acts rapidly upon the embryo, with some tissues more susceptible than others, and demonstrates that short-term exposure to the NA/EE-mixture in zebrafish induces significant defects in embryonic development and movement (n ≥ 4 for DMSO controls; n ≥ 5 for NA/EE-mixture).

### Quantification of the dose of the drug that reaches the embryo

**Figure 4:**
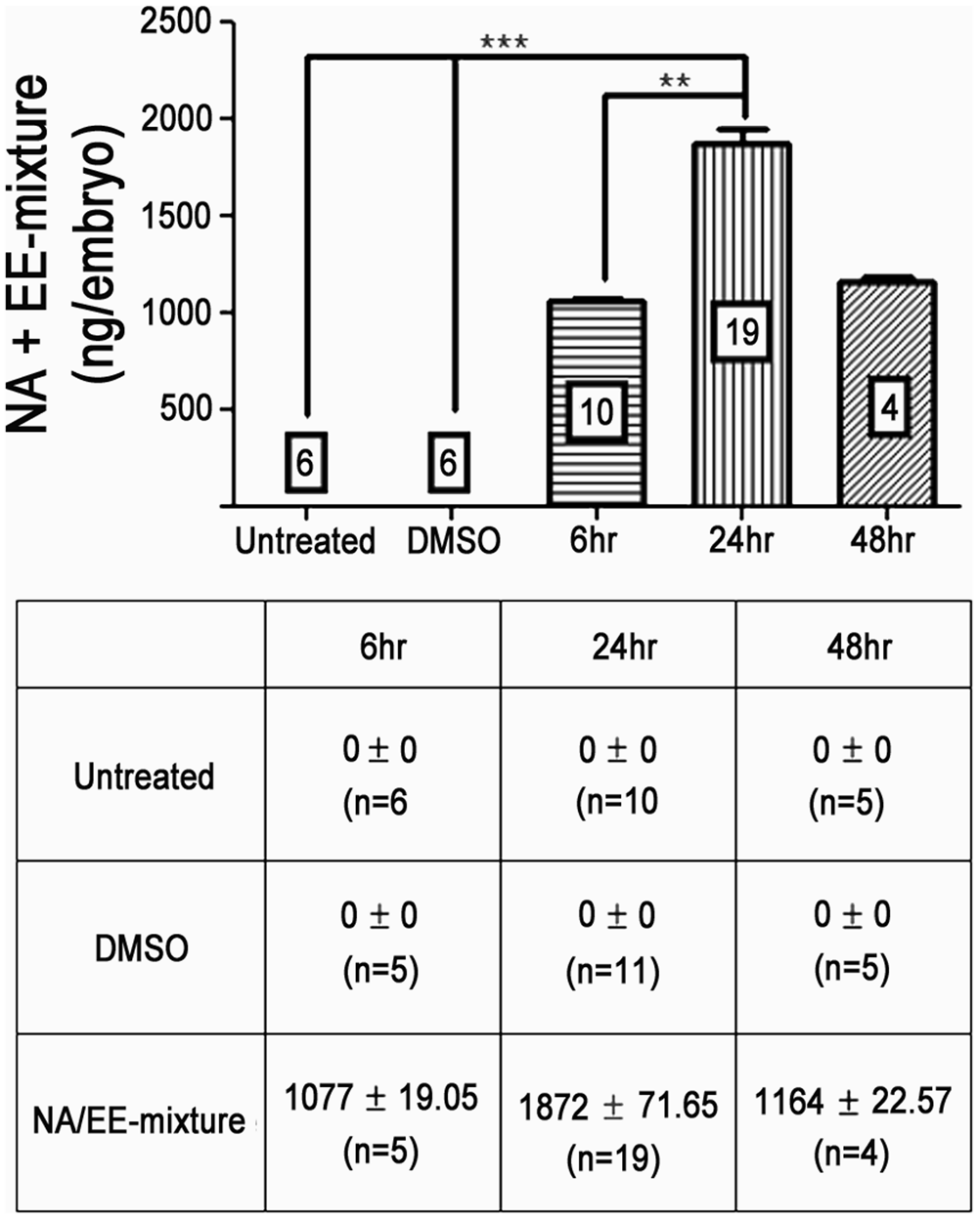
The concentration of NA within the developing zebrafish embryo exposed to NA/EE-mixture peaks at 1.8 µg/embryo at 24 hours incubation. (**A**) Graph depicting the concentration of NA in Untreated (water only) and DMSO controls compared to the concentration of NA found in zebrafish embryos exposed to NA/EE-mixture for 6 hr, 24 hr and 48 hr. (**B**) Table showing average concentration of NA (±SEM) at each timepoint in Untreated and DMSO controls alongside NA/EE-mixture exposed embryos. This data was used to present the graph in (**A**). At all timepoints there was 0 ng/mL (±0 ng/mL) NA in both the untreated and DMSO controls. At 6 h exposure, there was an average of 1077 ng/mL (±19.05 ng/mL) NA, peaking at 1872 ng/mL (±71.65 ng/mL) NA at 24 hr exposure. By 48 hr the NA concentration had started to decrease to 1164 ng/mL (±22.57 ng/mL). n numbers are stated in the boxed areas within the graph (A) or as (n = x) in the table (**B**). Statistical significance was analysed using Kruskal-Wallis and Dunns post hoc test. Graph and table represent mean concentration of NA per embryo ± SEM. **p < 0.01; ***p < 0.001.

Next, we used LC-MS/MS Mass Spectroscopy to determine the concentration of the drug in the embryo. For this analysis we focused upon NA as levels of EE were consistently below detection rates. Embryos at 24 hpf were placed in the NA/EE-mixture (12.5 μg/mL NA + 25 ng/mL EE), or in DMSO or in water (untreated) for 6 hr, 24 hr or 48 hr. Embryos were rinsed in water and then frozen before LC-MS/MS Mass Spectroscopy analysis. We found that the level of NA in the embryos was 1 µg/embryo within 6 hr of treatment, peaking at 1.8 µg/embryo at 24 hr and subsiding to 1.2 µg/embryo at 48 hr (Fig. 4). This data indicates that NA can accumulate in embryonic tissue for at least 24 hrs.

### NA/EE-mixture exposure increases cell death and reduces cell proliferation throughout the embryo

**Figure 5:**
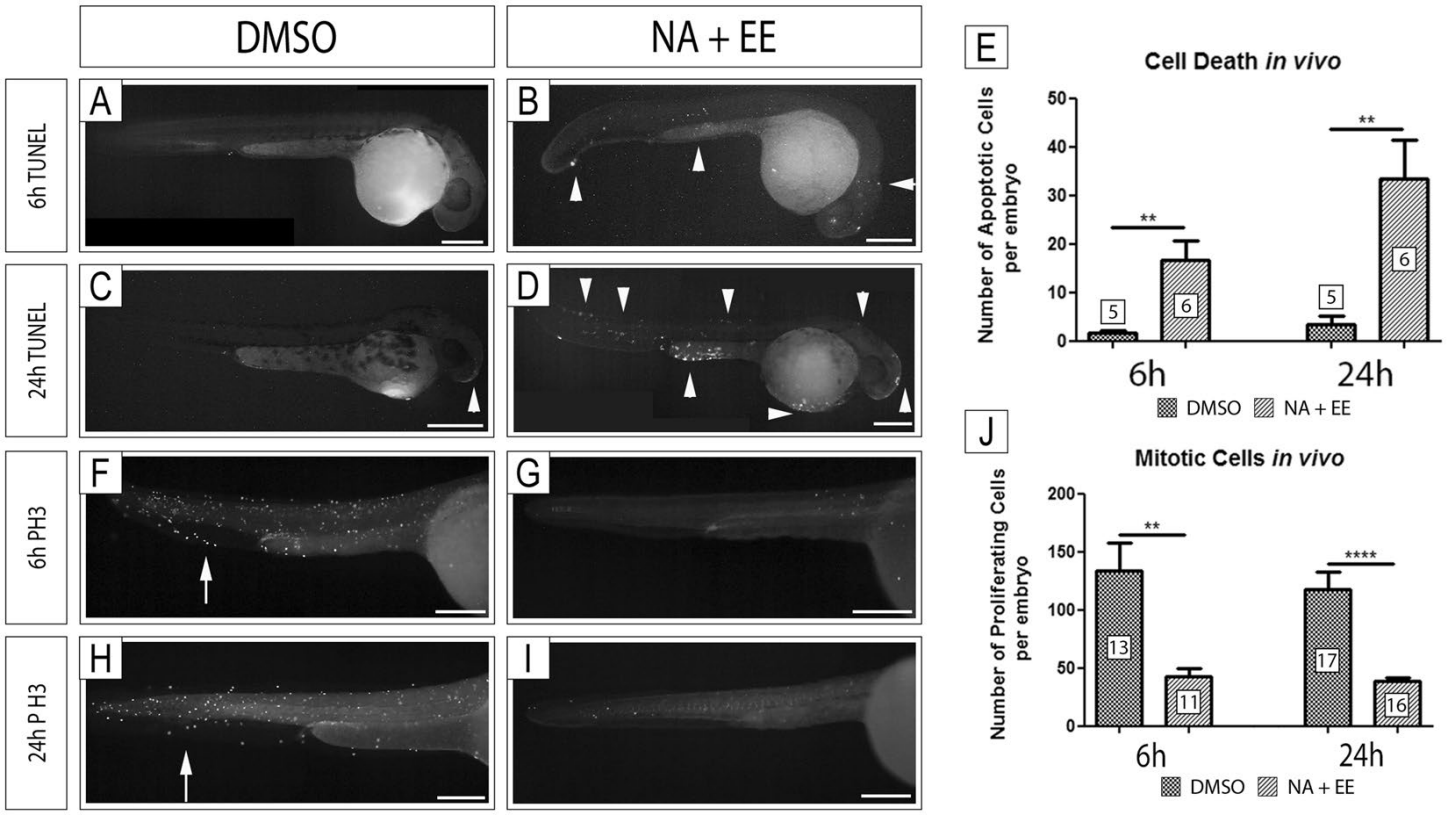
Cell Death is increased and Cell Proliferation decreased in Zebrafish Embryos Treated with NA/EE-mixture Zebrafish embryos at 24 hpf were treated with either DMSO or NA/EE-mixture and fixed at 6 hrs or 24 hrs post-treatment. Cell death and cell proliferation analyses were assessed by TUNEL assay (**A**–**D**) or antibody staining with anti-Phosphohistone H3 (**F**–**I**), respectively. The number of apoptotic cells or cell undergoing mitosis were counted and compared between drug treated and DMSO controls. Proliferating cells were counted from the position of the yolk sac to the tail, not including the yolk sac or yolk extension. (**A**–**E**) Embryos treated with NA/EE-mixture present a higher number of apoptotic cells at 6 hours (**A**,**B** and **E**) and 24 hours (**C**–**E**). Apoptotic cells do not occur in specific regions of the embryos. White arrowheads denote examples of apoptotic cells, which outnumber those (if any) seen in DMSO controls. (**F**–**J**) Embryos treated with NA/EE-mixture present a significantly lower number of proliferating cells at 6 hours (**F**,**G** and **J**) and 24 hours (**H**–**J**). Proliferating cells are reduced in all regions of the embryos. White arrows indicate regions of proliferating cells, markedly reduced in treated embryos. Statistical significance was analysed using unpaired t test. Graphs represent mean ± SEM. **p < 0.01, ****p < 0.0001. Scale bars: 250 µm.

We next investigated if cell death was induced by the NA/EE-mixture in treated embryos to potentially explain the damage and phenotypes observed. In order to analyse cell death, we performed a TUNEL assay in embryos treated with NA/EE-mixture (12.5 μg/mL NA + 25 ng/mL EE), or DMSO and fixed at 6 hrs and 24 hrs after exposure (n ≥ 5 for each condition and time-point). At both time points cell death was increased significantly in embryos treated with the NA/EE-mixture (p < 0.01; Fig. 5A–E). The increased cell death was not localised to specific tissues, for example, just to the eye, pectoral fin or tail but was observed throughout the embryo, correlating with the decrease in overall body size as well as fin and eye size in the treated embryos.

We also investigated if cell proliferation changes occurred in embryos treated with NA/EE-mixture. We treated embryos at 24 hpf, and fixed and stained for Phospho-histone H3, a marker of mitosis, at 6 hr and 24 hr after exposure (n = 11 for 6 hr, n = 16 for 24 hr) and compared to DMSO controls (n = 13 for 6 hr, n = 17 for 24 hr). To ensure the consistency of the analyses, Phospho-histone H3 positive cells were counted from the position of the yolk sac to the tail, excluding yolk sac and yolk extension. We found a decrease in the number of mitotic cells at both 6 hr and 24 hr in embryos exposed to the NA/EE-mixture exposed embryos compared to controls (p < 0.01 at 6 h; p < 0.0001 at 24 hr; Fig. 5F–J). Similarly, to the cell death analyses we didn’t observe any regional variations in cell proliferation, but a general decrease in cell proliferation throughout the embryo in NA/EE-treated embryos.

### NA/EE-mixture exposure alters embryonic blood vessel patterning

**Figure 6:**
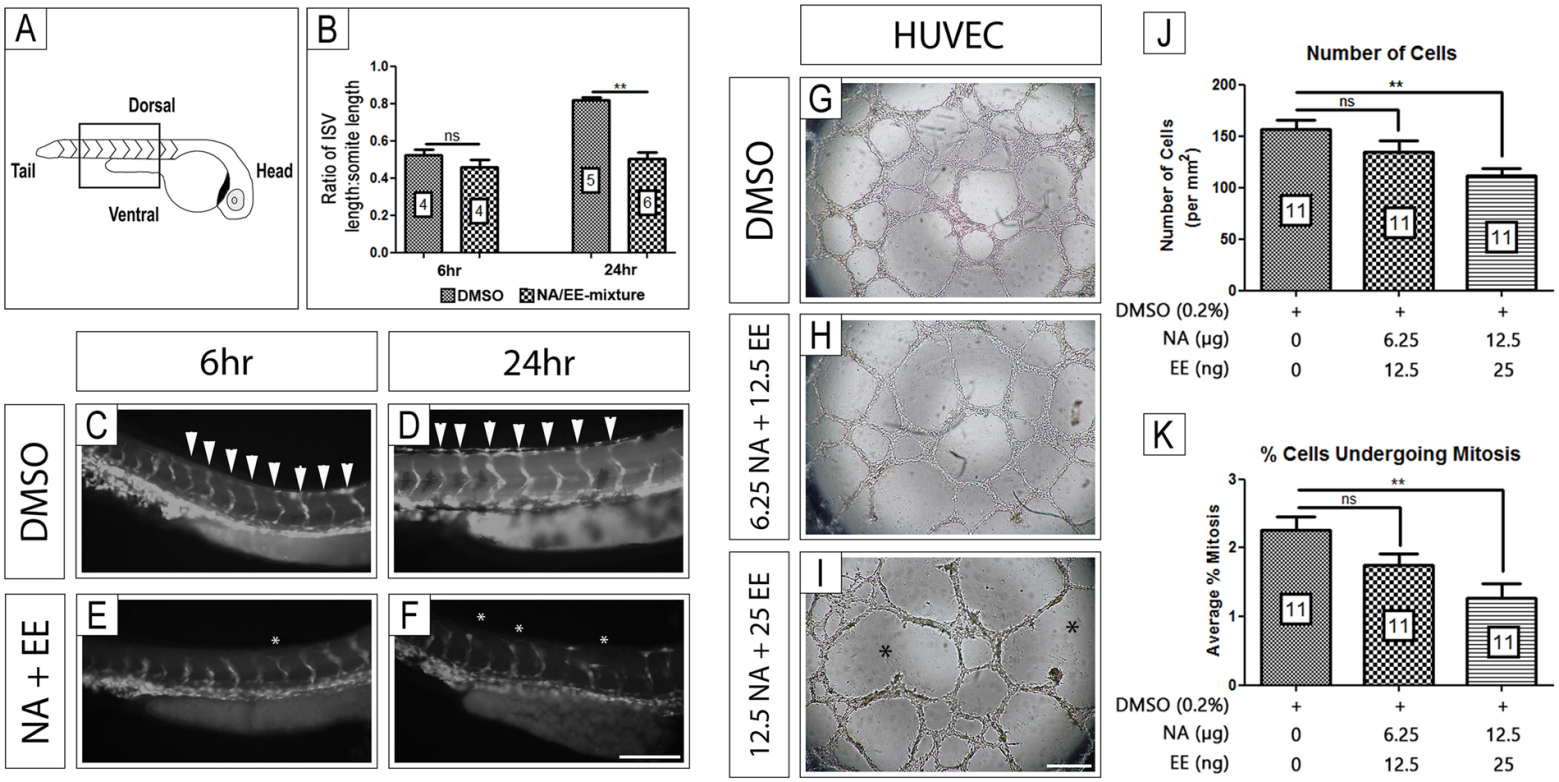
NA/EE-mixture exposure *in vivo* and *in vitro* causes vessel formation anomalies The effect of NA/EE-mixture on blood vessels was assessed by using zebrafish *fli1*:EGFP line and HUVEC culture. (**A**–**F**) *In vivo* growth of intersomitic blood vessels (highlighted by box in panel A) and patterning were analysed following treatment with either DMSO or NA/EE-mixture (NA 12.5 μg/mL and EE 25 ng/mL) for 6 or 24 hrs following treatment. (**B**) Ratio of intersomitic vessel length (ISV) to somite length 6 hrs and 24 hrs after treatment with DMSO (vehicle control) or NA/EE-mixture at 24 hpf. Statistical significance was analysed by Mann-Whitney test. (**C**,**D**) DMSO controls presented normal blood vessel growth and patterning when compared with untreated embryos (data not shown)^41^. (**E**,**F**) NA/EE-mixture treated embryos presented changes in growth and patterning of intersomitic vessels. (**G**–**I**) NA/EE-mixture disrupts the tube formation in HUVEC cultures in a dose-sensitive manner. (**J**,**K**) NA/EE-mixture reduces cell number and cell proliferation in a dose-sensitive manner in HUVEC cultures. Statistical significance was analysed using One-way ANOVA followed by Tukey’s test. Graphs represent mean ± SEM. **p < 0.01. Scale bar E: 100 µm; scale bar H: 500 µm. White arrowheads denote normal position and pattern of Intersomitic vessels. White asterisks indicate examples of misposition and mispatterning of vessels, including vessel fusions. Black asterisk denote avascular and poorly patterned areas of treated HUVEC cultures.

Previously we have shown that drugs such as thalidomide and some of its analogs^35,37–39^ as well as antiangiogenic agents such as Sunitinib^29,34^ cause damage to a range of tissues including the fins, otic vesicle and eyes through disrupting blood vessel formation. To investigate if blood vessel loss or patterning defects occur in zebrafish embryos following NA/EE-mixture exposure we used the transgenic *fli1*:EGFP reporter line of zebrafish embryos^40^. These embryos express enhanced Green Fluorescent Protein (EGFP) in blood vessels, which can be visualised live and *in vivo* when placed under a fluorescence light source^30,38,40^. *fli1*:EGFP zebrafish embryos at 24 hpf were incubated with either DMSO (n = 18 for 6 hr, n = 21 for 24 hr) or the NA/EE-mixture (12.5 μg/mL NA + 25 ng/mL EE) and their intersomitic vessels (ISV) imaged at 6 and 24 hours after exposure (Fig. 6A; n = 19 for 6 hr, n = 18 for 24 hr). ISVs are easy to visualise at the stages assessed and, because they develop in a rostral-caudal gradient along the embryo, this enables the effects of compounds on formed vessels, vessels beginning to form and areas where angiogenesis is yet to be initiated, to be determined in the same embryo (Fig. 6).

Embryos incubated with DMSO (Fig. 6C,D) displayed complete dorsal vessel anastomosis 24 hours after exposure (48 hpf), comparable to untreated wild-type embryos^40^. In contrast, incubation with the NA/EE-mixture (Fig. 6E,F) caused some mispatterning of vessels within 6 hr exposure (Fig. 6E) and misplacing, mispatterning and stunting of intersomitic vessel outgrowth throughout the spine of the embryo 24 hr following drug exposure (Fig. 6F). Quantification of intersomitic vessel outgrowth demonstrated no outgrowth deficit at 6 hr but significant reduction in outgrowth by 24 hr (Fig. 6B).

Because the intersomitic vessel defects could be secondary, for example due to changes in somite formation which have been shown to be the cause of vessel positioning changes in Notch signalling pathway mutants^41^ we next studied the effect of NA/EE-mixture exposure directly using *in vitro* cultures of cells from a human umbilical vein endothelial cell line (HUVEC). HUVEC cells form networks of endothelial cell tubes which branch and provide a method to ascertain the effects of direct application of compounds to blood vessels^32,38^. Application of the NA/EE-mixture to newly plated HUVEC cells before the HUVEC cells have formed endothelial cell tubes caused changes to the number of branches of endothelial tubes in a dose-sensitive manner (Fig. 6G–I). Cell proliferation and cell number also was decreased in a concentration dependent manner (Fig. 6J,K). Despite the number of endothelial cells and their proliferation rates being reduced at each concentration, they were still able to form patterned, branched, vascular networks, though bigger gaps are seen between the endothelial tubes. This suggests vessels can form in the presence of the NA/EE-mixture but endothelial cell proliferation and vessel branching is perturbed.

### NA/EE-mixture exposure affects nerve patterning and outgrowth *in vivo* and *in vitro*

We have demonstrated that the NA/EE-mixture treated embryos exhibited movement loss within 1 hr of exposure (Fig. 3). In addition, embryos also exhibit bent spines, following 24 hrs exposure. We therefore investigated the effect of the NA/EE-mixture upon the nervous system. First, we focused on neurite outgrowth in embryos treated with the NA/EE-mixture. Embryos at 24 hpf treated with either DMSO or the NA/EE-mixture (12.5 μg/mL NA + 25 ng/mL EE) were fixed at 6 hr and 24 hr then stained with an anti-neurofilament antibody to analyse nerve patterning.

**Figure 7:**
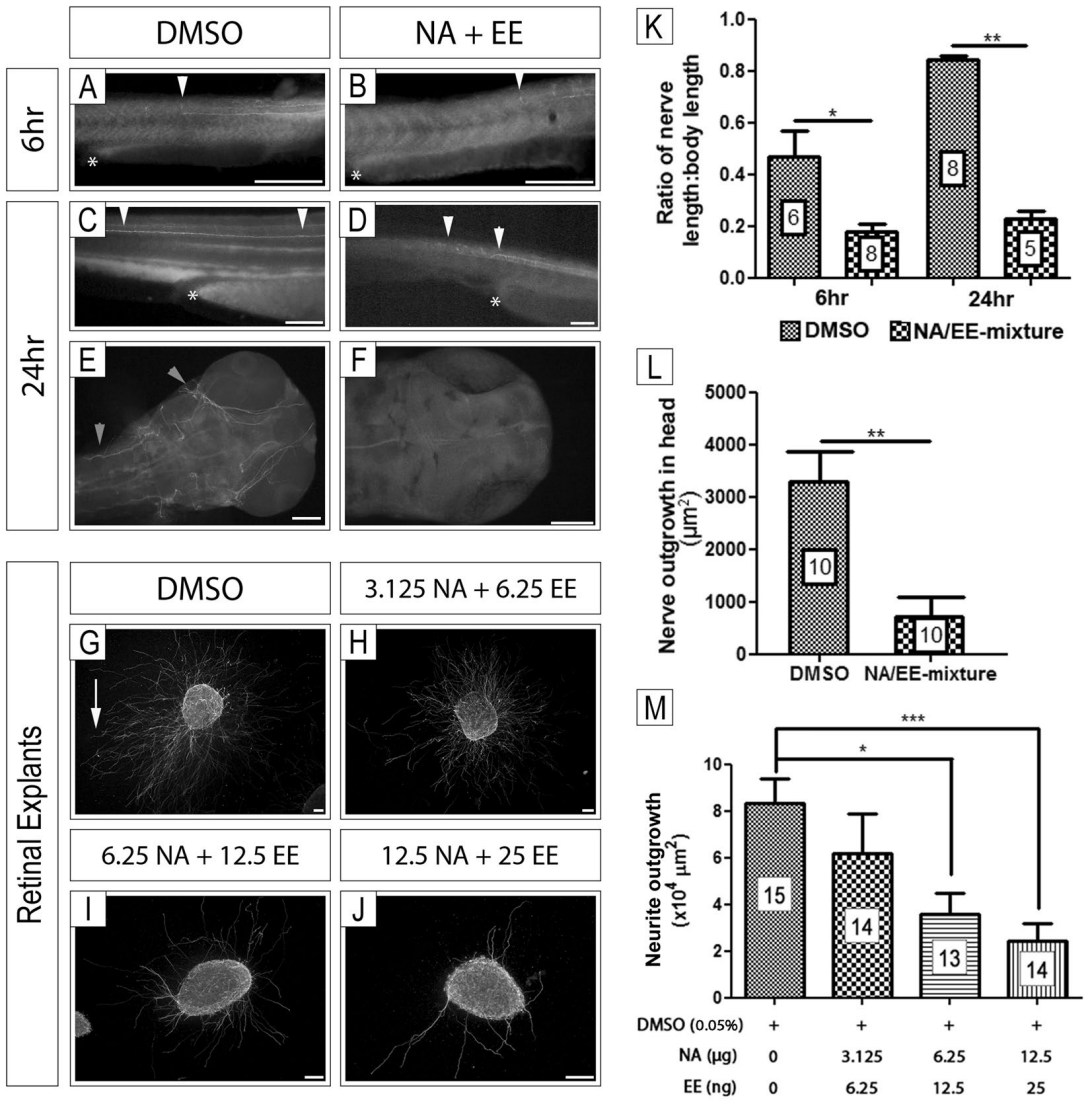
*In vivo* and *in vitro* neuro-inhibitory effects of NA/EE-mixture exposure NA/EE-mixture effects on zebrafish nerve outgrowth and patterning. Embryos were treated at 24 hpf with either DMSO (**A**,**C** and **E**) or NA/EE-mixture (**B**,**D** and **F**) and fixed at 6 hours and 24 hours. Embryos incubated with DMSO present normal nerve outgrowth and patterning. Compared to DMSO controls, embryos treated with NA/EE-mixture presented fasciculation defects. White asterisk denotes end of yolk sac. White arrowheads denote position of major nerve tract in spinal cord; at 24 hr nerve tract is stunted and defasciculated in treated embryos, compare arrowheads in C and D. Nerves are missing in the head of treated embryos (**F**) compared with control embryos, grey arrowhead denotes axon projections (**E**). Direct effect of NA/EE-mixture on nerves was assessed through retinal explants culture exposed to DMSO and NA/EE-mixture over different concentrations; 0.05% DMSO (**G**), NA 3.125 μg/mL + EE 6.25 ng/mL (**H**), NA 6.25 μg/mL + EE 12.5 ng/mL (**I**) and NA 12.5 μg/mL + EE 25 ng/mL (**J**) treatment. (**K**) Ratio of nerve length to body length is reduced in treated embryos at 6 hpf and 24 hpf as is the ratio of nerve outgrowth (**L**) in the head following treatment at 24 hpf. Statistical significance was analysed through Mann-Whitney test. (**M**) Neurite outgrowth in retinal explants was decreased significantly following NA/EE-mixture exposure at NA 6.25 μg/mL + EE 12.5 ng/mL (**H** and **M**) and NA 12.5 μg/mL + EE 25 ng/mL (**J** and **M**). White arrow denotes an example of an axon projection. Statistical significance was analysed using Kruskal-Wallis test with Dunn’s post-hoc test. Graphs represent mean ± S.E.M. ns, p > 0.05; *p < 0.05; ***p < 0.001. Scale bars: 100 µm.

Embryos treated with NA/EE-mixture (n = 9 for 6 hr and n = 16 for 24 hr; Fig. 7B,D) presented defasciculation of axons in the developing spinal cord and shortening of axonal outgrowth. In DMSO treated embryos axons can be seen extending through the spinal cord to midway through the spine after 6 hrs and throughout the spine by 24 hr exposure (Fig. 7A,C). In contrast in the NA/EE-mixture treated embryos axons had not extended to the midpoint of the spine by 6 hrs and failed to innervate the tail region of the embryo by 24 hr (Fig. 7B,D). Quantification of nerve length relative to overall body length indicated significant nerve length reduction from 6 hrs following drug exposure (Fig. 7K). In the developing head of the embryo nerves are also disorganised, mispatterned and defasiculated when compared to embryos treated with DMSO (Fig. 7E,F)(n = 7 for 6 hr and n = 12 for 24 hr). Total nerve outgrowth in the head also was reduced significantly in treated embryos (Fig. 7L).

To confirm whether the neuroinhibitory action observed in the embryos was direct we tested the effects of the NA/EE-mixture exposure on neurite outgrowth directly using an *in vitro* mouse retinal explant assay^32,42–44^. Retinas were dissected from E14.5 C57BL/6 J WT mice and cultured in DMSO or in a range of concentrations of the NA/EE-mixture (3.125 μg/mL NA + 6.25 ng/mL EE; 6.25 μg/mL NA + 12.5 ng/mL EE; and 12.5 μg/mL NA + 25 ng/mL EE; Fig. 7G–J). After 48 hr, the cultures were fixed and stained with a neuron-specific anti–β-tubulin antibody and the area of neurite outgrowth from the cultures quantified.

We found that the drug has a dose-dependent inhibitory effect on neurite outgrowth. Treatment with 3.125 μg/mL NA + 6.25 ng/mL EE had no significant effect on neurite outgrowth (n = 14; Fig. 7H,M) when compared to DMSO (vehicle) controls (n = 15; Fig. 7G,M). However, the extent of neurite outgrowth from retinal explants exposed to 6.25 μg/mL NA + 12.5 ng/mL EE (n = 13; Fig. 7I,M) and 12.5 μg/mL NA + 25 ng/mL EE (n = 13; Fig. 7J,M) was decreased significantly compared to DMSO controls. These findings demonstrate that the NA/EE-mixture can inhibit nerve outgrowth when directly applied to *in vitro* nerve explants and can also cause axonal outgrowth defects *in vivo*.

## Discussion

We have demonstrated that a mixture of NA/EE (the components of Primodos), can cause developmental anomalies when directly applied to zebrafish embryos. The compound acts in both a dose-dependent and time sensitive manner, with early exposure causing more damage than later exposure. Damage also is extremely rapid. Within 1 hr of drug exposure at 24 hpf, embryos displayed significantly reduced movement, and, within 4 hrs of exposure, obvious morphological defects. Using *in vitro* assays utilising human HUVEC cells and mouse retinal explants we found that the NA/EE-mixture directly impairs blood vessel pattern formation and nerve outgrowth. These findings demonstrate that the components of Primodos are potentially teratogenic, affecting the development of a wide range of zebrafish organ systems *in vivo* and further provides evidence that these components can affect the development of mammalian tissues *in vitro*.

Previous work in zebrafish embryos have studied the effects of prolonged EE or NA exposures (ie: continously from 0 hpf throughout embryonic development and up to adulthood). Such work has shown EE can disrupt forebrain neural patterning^45,46^ and cause defects including uninflated swimming bladder, body axis curvature and, pericardial and yolk sac oedemas^47^, cause embryonic malformations in resulting offspring^48^ and affect fertility^49^. In additon some progestins, including NA, have been shown to misregulate enzymes, like aromatase, essential for biosynthesis of estrogens in radial glial cells in the brain^24^. However, human embryos exposed to Primodos used as a pregnancy test would not be continously exposed to elevated hormone levels as done in these studies. We therefore exposed embryos to single doses of a combined NA/EE-mixture (in a ratio equivalent to that seen in Primodos) for 1–24 hr at 6, 24, 48 or 72 hpf, (a time period approximate to the developmental stage in human development that hormone pregnancy tests would likely be used) and demonstrated that this also caused damage, although at higher concentrations (12.5 μg/mL NA + 25 ng/mL EE in our experiments versus 0.34 mg/L NA; 14.8 ng/L EE and 1–20 ng/L EE in the prolonged exposure studies). Furthermore we have shown that the of NA/EE-mixture induces damage rapidly, affecting movement of 24 hpf embryos within 1 hr of application and morphological damage within 4 hrs. The difference in concentrations required to induce embryonic damage in this study compared to previous work^45,46,48,49^ likely reflects the shorter time course of drug application in our study. In keeping with this idea, we have shown that the drug accumulates in embryos over time. Thus, prolonged exposure will likely result in higher concentrations within the embryo as development proceeds.

The dose we chose to investigate the action of the NA/EE-mixture upon embryonic development (12.5 μg/mL NA + 25 ng/mL EE) was determined from carrying out a dose response analysis (Fig. 1). At the 24 hpf developmental timepoint lower doses had no effect, whilst higher doses caused severe damage or death. How does the dose used in our study compare with the doses used in humans? The peak of NA in human plasma averages 18.3 ng/mL, or 0.0183 μg/mL, when a 1 mg dose is taken^50,51^ and averages 26 ng/ml in human plasma with 1–2 hours of administration with a 5 mg dose^28^. Considering that when used as a pregnancy test, the dose of Primodos taken was 10 mg, it is expected that the circulating NA would be higher. Moreover, given the normally elevated levels of progesterone and oestrogen during pregnancy, (from 10–54 ng/mL and 486–1615 pg/mL respectively)^52–54^ the use of a synthetic progesterone based hormone will result in a total higher concentration in pregnant versus non-pregnant women. We used Mass spectroscopy to measure the levels of NA within the zebrafish embryos and found that in just 6 hrs the concentration was 1 µg/embryo and the concentration peaked at 1.8 µg/embryo after 24 hr incubation. The doses we are using are higher than the plasmatic dose seen in humans after Primodos exposure. However, we do not know the receptor specificity or transport ability of these synthetic human hormones in zebrafish, which could be significantly different. Thus, it is difficult to extrapolate from our work what would be teratogenic dose in humans. Moreover, in human plasma the half-life of NA, a synthetic progestogen, is much longer (up to 9 hours)^28,55^ than endogenous progesterone (reportedly 5 mins)^56^. Even though the drug would then dilute throughout the blood plasma and likely be metabolised in the maternal liver, a study investigating NA/EE uptake in early human pregnancies showed levels of NA in the maternal blood plasma were elevated for up to 48 hrs after exposure, however, levels of NA/EE in the embryos were not described^57^. As there is little to no metabolic liver function in early embryogenesis^58,59^ it is possible that the drug concentration will accumulate and build up to high levels in the human embryo over time, as we have observed in the zebrafish embryos.

The zebrafish possesses progesterone and oestrogen receptors and their expression patterns show potential roles in brain, ovary, testis, epidermis, head, trunk, hatching gland and pectoral fin buds^60–62^; tissues we have reported damage to following treatment with NA/EE. Oestradiol has also been shown to be involved with cardiac and liver development and also in embryonic heart rate regulation, changes to which could result in embryonic growth problems^63,64^. As well as this, progesterone signalling has been detected in the developing pancreas, central nervous system, muscle, and pectoral fin buds^65^. This highlights the multiple embryonic tissues that the sex steroids can potentially influence. Whether this is via a direct, receptor mediated interaction or paracrine mechanisms remains unclear^66^.

Oestradiol also is involved with vitellogenin production in embryos which in turn goes on to form part of the yolk sac, a source of nutrients for the developing embryo^67^. If development of the yolk sac is abnormal, this may infringe upon nutrient supply to the embryo and may lead to additional congenital abnormalities. We see yolk sac changes as well as damaged tissues and changes in cell death and proliferation following exposure to NA/EE-mixture. Progesterone also is known to be metabolised into corticosteroids such as cortisol by the foetal kidney and adrenal gland^68^, which if found at high levels has teratogenic effecst in zebrafish^69,70^, and mammals such as sheep^71^. This raises the possibility that exogenous progesterone, and synthetic forms like NA, might metabolise into these teratogenic compounds, and thus cause developmental defects within the embryos.

We have shown the NA/EE-mixture has effects on cell death and cell proliferation in the zebrafish embryo and which could explain the reduced length and some of the tissue damage. We also show nerve damage which could explain the reduced embryo movement and possibly reduced embryonic length. Work in mouse and chicken embryos indicate loss of nerves in developing limbs can cause reduced outgrowth of the limb as a whole but not patterning defects/damage such as loss of specific bony elements^72^. This indicates nerve inhibition is more likely to exacerbate damage already caused by some other factor. We also demonstrate vascular changes *in vivo* in zebrafish embryos and also *in vitro* in HUVEC assays. Several known teratogens including thalidomide and valproate^37,38^ as well as anticancer antiangiogenic drugs^34^ are thought to cause embryonic damage through vessel inhibition. However precisely how loss of vessels results in embryonic damage remains unclear and further work is needed. Recent work has shown that progesterone and oestrogen regulates expression of vascular regulators including VEGF and angiopoietin in human^73^ and in primate endometrium^66^. Taken altogether, this suggests that the NA/EE-mixture can induce embryological defects through a range of mechanisms including, impaired nerve growth and angiogenesis, elevated cell death and impaired cell proliferation.

The range of damage seen in alleged victims of Primodos exposure has not been fully documented but includes damage in the extremities of the body including fingers and face, ears and CNS/brain^6,7,11,13,14,74,75^. We have found that the range of defects induced by the NA/EE-mixture depends on the stage of development when the embryo is exposed to the drug (Fig. 2). Exposure to very early stage embryos (6 hpf) causes much more severe damage than seen following exposure to late embryonic stages (72 pf; Fig. 2). The Primodos pregnancy test would have been taken over a large range of time in embryogenesis; women intending to become pregnant would likely have taken the test shortly after the first missed period, whilst women not expecting to be pregnant may wait longer. The time point at which the pregnancy test was taken would determine which developmental process has the most potential to be affected and thus result in a wide variety of potential defects, as seen in alleged Primodos survivors.

It is, of course, difficult and dangerous to directly compare drug action/s between species. Nevertheless, our data demonstrates accumulation of the drug in the embryo, which does not decrease for some time, and leads to rapid embryonic damage. From other animal models of drug-induced teratogenesis, for example thalidomide exposure, higher doses, than used in humans, are required to reciprocate the damage seen in humans due to differences in applications, uptake and metabolism^37,76–78^. We have used a NA/EE-mixture composed of synthetic human progestogen and oestrogen compounds, but whether the zebrafish progesterone and oestrogen receptors bind these compounds with a similar affinity to human progesterone and oestrogen receptors is unclear, and this might contribute to why high doses of the NA/EE-mixture were needed to see zebrafish embryonic damage. Clearly more work is required in mammalian species to confirm our findings.

In summary, we have shown that a NA/EE-mixture causes a range of damage to zebrafish embryos in a dose and time sensitive response to the NA/EE-mixture, which can accumulate in the embryo. The effects on the embryo are rapid, demonstrating that a short elevation in concentration is enough to induce damage. The concentration we have used is higher than human plasma concentrations of NA. However, given differences in drug application, absorption, metabolism and possibly species differences in sensitivity of receptors, caution must be applied when extrapolating drug concentrations across species. Moreover, as we have demonstrated NA accumulates in the zebrafish embryo this may also occur in mammalian embryos and result in increased intraembryonic concentrations and, consequently, damage.

Taken altogether this work underlines the need for further, detailed research in mammalian species to determine the actions of the components of Primodos. Our studies in the zebrafish embryo has provided a starting point in understanding drug/compound action and determining the potential action of a drug/compound. This provides the basis and reasoning for further and more detailed studies in mammalian species to understand the full impact on mammalian embryos and the molecular pathways affected.

## Materials and Methods

### Compounds

Primodos (which is no longer available) was made up of 10 mg Norethisterone acetate (NA) and 0.02 mg Ethinyl estradiol (EE). Norethisterone acetate (NA; Sigma Aldrich) and Ethinyl Estradiol (EE; Sigma Aldrich) were dissolved in DMSO (Sigma-Aldrich) at stock concentrations of 25 mg/mL and 1 mg/mL, respectively. The stock solutions were dissolved in distilled water to reach the final, working concentrations which were applied at a ratio between NA and EE equivalent to the dose given to women (500:1).

### Zebrafish embryology and drug treatment

Adult zebrafish were bred and maintained as described previously^35,41^. Embryos collected from the tanks were kept in water to reach the desired developmental stage for drug treatment. Embryo stage is given in hours post fertilisation (hpf). Wildtype (WT) zebrafish embryos at 24 hpf, 48 hpf and 72 hpf were exposed to mixtures of NA/EE (in ratios equivalent to Primodos) under different concentrations or DMSO only (0.2%). Drug testing and analysis were carried out as described previously^30,32,34,35,41^. Briefly, embryos were hand dechorionated and exposed to the drugs or DMSO. For phenotypical analyses, embryos were fixed in 4% Paraformadehyde (Sigma-Aldrich) in 1x PBS at 96 hpf and for gene expression analyses, cell death and immunohistochemistry, embryos were fixed in 4% paraformaldehyde at 6 hr and 24 hr following treatment.

*fli1*:EGFP zebrafish embryos (obtained from the Zebrafish International Resource Center) were used to analyse the effects of the NA/EE mixtures on blood vessel growth using previously published protocols^30,32,34,35,40,41^. All animal research was licensed, approved and carried out following guidelines issued by the UK Home Office and University of Aberdeen Ethics Review Committee.

### Whole-Mount immunohistochemistry

Whole-mount antibody staining was carried out as described previously^41^ with minor modifications: embryos at 48 hpf or older underwent bleaching (KOH Peroxidase) for 20 minutes (48 hpf) or 30 minutes (72 hpf) and permeabilisation was performed with either ice-cold Acetone (Sigma-Aldrich) for 8 minutes at −20 °C for embryos up to stage 24 hpf or Collagenase A solution for 35 minutes (48 hpf) or 45 minutes (72 hpf). To label the nerves and proliferating cells, embryos were stained with 3A10 antibody (1:250; Developmental Studies Hybridoma Bank) or anti-Phosphohistone H3 antibody (1:150; Millipore 06–570) respectively.

### Cell death analyses

Cell death analyses were performed using the *In Situ* Cell Death Detection Kit (TUNEL-Roche) as described previously^79^. Briefly, tissues were fixed overnight in 4% PFA and washed in PBS for 5 minutes. Embryos were dehydrated in serial washes of ethanol and permeabilised with ice cold acetone for 10 minutes at −20 °C. Embryos were rinsed in PBS and washed again in PBS for 30 minutes. Embryos underwent a second permeabilisation with 0.1% Triton, 0.1% Sodium citrate in PBS for 15 minutes and rinsed twice for 5 minutes each in PBS. Embryos were incubated a mixture of 450 μL of solution 1 (TUNEL kit) and 50 μL of solution 2 (TUNEL kit for 60 minutes at 37 °C, rinsed 3 times in PBS for 10 minutes each and stored in PBS.

### Cell culture

HUVEC cells (Lonza #C2517) were cultured in endothelial cell medium in 96 well tissue culture plates or with use of a HUVEC angiogenesis kit for vascular tube formation as described previously^38^. Cells were exposed to three concentrations of NA/EE mixture or 0.02% DMSO as a control. The cells were incubated for 18 hr at 37 °C, fixed in 4% PFA for 1 hour, stained with antibodies for Phosphohistone H3 (Millipore; 1:500) and mounted in Vectashield with DAPI (Vector Laboratories).

### Retinal explant cultures

Experiments were performed using wild-type E14.5 C57BL/6 J mouse embryos from an in-house breeding colony. Retinal explants were prepared and analysed as described previously^32,43^. Results are the mean (±SEM) from two independent experiments for each condition.

### Imaging and analyses

Imaging of embryos was performed using a Nikon SMZ1500 fluorescent stereomicroscope with a Nikon DS-5 digital camera or using a Zeiss Axiophot epifluorescent microscope with a Nikon DXM1200 camera. Images of HUVEC cultures were captured on a Nikon Eclipse TS100 microscope fitted with a DS-Fi1c camera with NIS-Elements D software and images of retinal explants were captured using a Nikon SMZ1500 microscope and DXM1200 camera with ACT-1 software. Data were analysed using Adobe Photoshop and Image J. Quantification of embryonic intersomitic vessel outgrowth and nerve outgrowth between treated and control conditions was measured using Image J. To take into account differences in embryo length results are displayed as a ratio between nerve or vessel length to body or somite length respectively. Statistical analyses were conducted using Prism 5.0 (GraphPad Software, La Jolla, CA) or SPSS 20.0 (SPSS Inc., Chicago, USA). Statistical significance was assessed using two-tailed unpaired Student’s *t*-test, Mann-Whitney *U*-test, One-way ANOVA or Kruskal-Wallis test followed by Tukey’s or Dunn’s test respectively. Error bars represent standard error of the mean.

### Video imaging and analyses

Zebrafish embryos were placed in wells of a 24-well plate from 24 hpf and treated with DMSO or NA/EE-mixture for 1–24 hr. No more than 5 embryos were placed in one well. The embryos were imaged using time-lapse recording on NIS-elements D software, captured at one image per 10 m/s. Each embryo was analysed individually for the number of movements in 2 minutes, then the average number of movements per embryo per minute was calculated. A Mann Whitney test was used for statistical analysis.

### Determination of Norethisterone acetate (NA) concentration in zebrafish embryos by HPLC-MS/MS

Norethisterone acetate (NA) levels were determined using a rapid LC-MS/MS assay. NA was dissolved in water at a concentration of 1 mg/mL and stored in aliquots at −20 °C. Quality control samples were prepared in water/methanol (50/50) at 10, 85 and 175 ng/mL NA and stored at −70 °C. Daily, NA was diluted in water/methanol (50/50) to give calibration standards in the range 6.25–200 ng/mL.

Embryos were exposed to the NA/EE-mixture for the appropriate time period and then rinsed in 10 ml water three times to remove excess solution. Embryos were stored individually in 100 µl of water and frozen for analysis. Individual embryos in 100 µL of water were homogenised by sonication and the resulting solution diluted 1:10 with water/methanol (50/50) and following centrifugation at 14800 rpm at 4 °C, 5 µL was injected onto the chromatograph. Chromatography was performed on a Thermo Surveyor (Thermo Scientific, UK) system using a 150 × 2.1 mm ACE 3 µ C18 column (Hichrom, UK) maintained at 50 °C. NA was resolved using isocratic elution with a mobile phase composition of 15% water/85% methanol (both containing 0.1% formic acid). The flow rate was 200 µL/min and the samples were maintained at 4 °C in the autosampler. NA eluted at 3.34 minutes and the total run time was 3.8 minutes.

A Thermo TSQ Quantum triple quadrupole mass spectrometer was used in positive electrospray ionisation mode for the detection of NA. Quantification was performed using multiple reaction monitoring (MRM) scan mode using the following transitions: *m/z* 341.2–91.1 at collision energy 35 V and *m/z* 341.2–109.1 at collision energy 26 V. Flow injection analysis was used to optimise the MS/MS conditions as follows: spray voltage 4000 V, sheath gas pressure 20, auxiliary gas pressure 35, capillary temperature 375 °C, skimmer offset −14V and collision pressure 2.0 mTorr. Instrument control and peak integration and quantification were performed using Thermo Xcalibur software (v. 3.0). Weighted least squares linear regression with a weighting factor of 1/X^2^ was used to quantify NESA concentrations in unknown samples by comparison of peak areas with those obtained from a multi-level calibration standard curve. The LLOQ for the assay was 1 ng/mL and the intra and inter-assay variations were determined to be <3% and <6% respectively.

### Ethical approval

All experimental protocols and procedures were *approved by* the University of Aberdeen Ethical Review Panel and is fully licensed by the UK Home Office.
